# Development and evaluation of a rapid analysis for HEPES determination in ^68^Ga-radiotracers

**DOI:** 10.1186/s13550-018-0449-6

**Published:** 2018-10-23

**Authors:** Sarah Pfaff, Tina Nehring, Verena Pichler, Jens Cardinale, Markus Mitterhauser, Marcus Hacker, Wolfgang Wadsak

**Affiliations:** 1 Division of Nuclear Medicine, Department of Biomedical Imaging and Image-guided Therapy, Medical University of Vienna, Waehringer Guertel 18-20, A-1090 Vienna, Austria; 2 Department of Inorganic Chemistry, University of Vienna, Vienna, Austria; 3 Ludwig Boltzmann Institute Applied Diagnostics, Vienna, Austria; 4 CBmed – Center for Biomarker Research in Medicine, Graz, Austria

**Keywords:** HEPES, Gallium-68, ^68^Ga-radiotracer, TLC, HPLC, Quality control, PET

## Abstract

**Background:**

HEPES is a favorable buffer for ^68^Ga-complexations in radiochemical laboratories. The drawback of this buffer is its prescribed limit of 200 μg per recommended application volume in the final formulation. Currently, a TLC test according to the European Pharmacopoeia (Ph. Eur.) has to be performed for quantification, but this analysis suffers from low reliability and reproducibility and is based on a subjective, semi-quantitative visual evaluation. In this study, the TLC method according to the Ph. Eur. and two literature-known HPLC assays for HEPES quantification were evaluated. Additionally, the development of an improved TLC method was performed.

**Results:**

The assay according to Antunes et al. provided a reasonable quantification of HEPES using HPLC. Additionally, a reliable and conclusive TLC method was developed, which facilitates quantitative analysis by means of a pixel-based evaluation. A comparison of those two methods with the Ph. Eur. TLC assay pinpoints the superiority of the HPLC as well as the new TLC assay. Furthermore, evaluation of HEPES contents using both TLC assays by 28 subjects supported the conclusion that the newly developed TLC method is clearly favorable.

**Conclusion:**

The TLC method according to the Ph. Eur. provides unsatisfactory results in terms of conclusiveness and reproducibility. In contrast, a reported HPLC assay showed valid results, with the drawback of high technical effort. An optimized alternative is provided by the improved TLC method described in this work that results in reliable outcomes and additionally offers quantitative analysis.

## Background

Radiopharmaceutical chemistry deals with short-lived nuclides, especially PET nuclides. Therefore, syntheses of PET radiotracers in this small niche of chemistry have to fulfill special prerequisites like short synthesis duration and minimal radiation exposure of the operator. In the first line, the duration of the semi- to fully automated production should be kept as short as possible to reduce the decay of the nuclide during the synthesis to a minimum. As a result, reaction conditions have to be optimized to meet the demands of rational PET tracer production.

In this article, the emphasis is put on one important parameter, the pH value that has to be taken into account, especially for complexations with radiometals. At a low pH, the coordinating moieties of the chelator can be protonated and therefore inactivated [1]. Focusing on gallium-68, the pH must be in a range of 3.5 to 5.5 due to the fact that gallium starts to form insoluble colloids and hydroxides at a higher pH that are not available for complexation [2, 3]. Correspondingly, pH buffering systems are necessary for a successful product formation. Appropriate buffers for ^68^Ga-labeling are supposed to be weak gallium-complexing agents that hinder hydrolysis to hydroxides and keep the metal in its active ionic state [4, 5]. Sodium acetate (NaOAc), sodium succinate, and 4-(2-hydroxyethyl)-1-piperazineethanesulfonic acid (HEPES) were reported as the most favorable buffers for ^68^Ga-labeling so far [6]. Nevertheless, the latter one was reported to result in better radiochemical yields for [^68^Ga]Ga-DOTATOC than the first ones at low precursor amounts [7]. Hence, HEPES should be the buffer of choice for clinical routine productions and especially scientific labeling approaches. However, the drawback of using HEPES is that the European Pharmacopoeia (Ph. Eur.) defines HEPES as an impurity that has to be below a specified limit [8].

One of the standard purification techniques prior to the final formulation of ^68^Ga-radiopharmaceuticals is the solid-phase extraction using reversed phase cartridges (e.g., SepPak C18 RP), followed by washing steps and finally the elution of the product. While in theory, the buffer should be removed completely during this procedure, in reality, those separations are rarely quantitative, and thus, the remaining content of the buffer has to be quantified prior to administration to patients [9]. For the development of a feasible analytical method, the chemical and physical properties of HEPES should be considered.

**Fig. 1:**
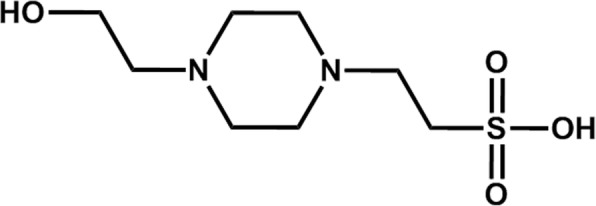
Chemical structure of HEPES (4-(2-hydroxyethyl)-1-piperazineethanesulfonic acid)

HEPES (Fig. 1) is one of the so-called Good’s buffers that is designed to meet several demands for biological experiments, for instance, a high water solubility being helpful for ^68^Ga-labeling in aqueous media [10]. Furthermore, this buffer should not interfere with analytical determination, e.g., UV/vis absorption by its intrinsic absorbance below 200 nm, which impedes the quantification at a low concentration range.

**Fig. 2:**
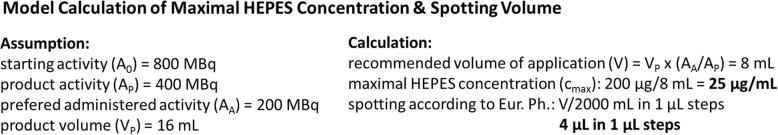
Model calculation of the product volume that has to be spotted on the TLC plate according to the Ph. Eur.

The Ph. Eur. sets a limit of 200 μg HEPES per maximum administration volume (*V*) (maximum recommended dose in milliliters). *V* depends on the total activity of the formulated product (*A*_*P*_) (see model calculation in Fig. 2). The higher the product activity is, the lower is the recommended administered volume (*V*) that contains the activity which is preferably administered to a patient (*A*_*A*_) and vice versa. Therefore, *V* depends on the starting activity (*A*_0_) which is limited by the capacity of the used ^68^Ge/^68^Ga-generator. As a result, *V* cannot be determined until the activity of the product is known. The Ph. Eur. stipulates a certain chromatographic testing method to determine the HEPES concentration of the final formulated product in comparison with a reference solution that requires *V* for its calculation. The prescribed thin-layer chromatography (TLC) analysis is performed by spotting the product solution next to a reference solution. The latter contains the limiting HEPES concentration (which depends on the product activity as explained above) in water. The spotted volume of each solution has to be the recommended administration volume *V* (in mL) divided by 2000 and spotted in 1 μL steps. After development of the TLC plate, HEPES is visualized by iodine staining and any spot must not be more intense than the reference solution, consequently containing only an equal or lower amount of HEPES.

This TLC assay was reported as neither reliable nor suitable and an improvement of this method would be highly advantageous [11]. As a result, a lot of effort has been made in the optimization and validation of an improved method for quantification of HEPES in radiopharmaceuticals by means of HPLC chromatography according to Kvaternik et al. and Antunes et al. [11–13].

The aim of this study was the comparison and evaluation of the TLC method, proposed by the European Directorate for the Quality of Medicine and Healthcare (EDQM), and established methods described in literature for quantification of HEPES in a complex matrix such as a formulated radiopharmaceutical. Finally, we aimed towards the development of a new, reliable, and reproducible method for quantitative HEPES analysis dealing with identified drawbacks as well as keeping the technical demands as low as possible.

## Methods

### Materials

All chemicals were used without further purification. The precursors, Pentixafor and PSMA-11 (both GMP grade), for ^68^Ga-labeling were provided from ABX (Radeberg, Germany). All other chemicals were obtained from Sigma Aldrich, Merck, Fluka, or Honeywell. Syntheses were performed using a synthesizer (GRP®) from Scintomics (Fürstenfeldbruck, Germany) with disposable single-use cassettes. TLC for radiochemical purity assessment was done by applying chromatography paper impregnated with silica gel (iTLC-SG plates) purchased from Agilent Technologies (Santa Clara, USA). HEPES testing was performed using silica gel 60 F245 20 × 20 cm aluminum plates and aluminum oxide plates 60 F254 20 × 20 cm obtained from Merck. HPLC analysis was performed using a VWR Hitachi Chromaster system (VWR International, Radnor, USA; pump 5160, column oven 5310, UV detector 5410) and separate scintillation detectors Gabi Star (2 × 2 mm) and Ramona from Elysia-Raytest (Straubenhardt, Germany) controlled by a clarity software from Data Apex (Prague, Czech Republic).

### Radiochemical synthesis of ^68^Ga-labeled PET tracers

Radiolabeling was performed according to a cationic purification protocol described elsewhere [14]. Briefly, the [^68^Ga]Ga^3+^-containing eluate was concentrated using a P-SH+ cartridge. Gallium-68 was eluted with NaCl solution (5 M) and transferred to a reactor containing either 40 μg Pentixafor or 2.5–10 μg PSMA-11 in 1.5 mL HEPES buffer (1 M). The reaction mixture was heated to 126 °C for 6 min and subsequently purified applying a SPE cartridge (C-18, Sep-Pak Light). The purified product was diluted with PBS buffer and transferred to a sterile vial. The product was tested for its HEPES content using HPLC and/or TLC aside from all other routine quality control tests.

### HPLC methods for HEPES quantification

The injection volume was 20 μL of reference solution and the product sample, respectively. All solutions were injected undiluted.

### HPLC method according to Kvaternik et al. [12]

An isocratic flow of 0.5 and 0.8 mL/min was set using an Obelisc N column (5 μm, 4.6 × 150 mm, Sielc, USA). The mobile phase consisted of 80/20 H_2_O/ACN (% *v*/*v*) under addition of 0.05% H_3_PO_4_. Absorbance was measured at 195 nm.

### HPLC method according to Antunes et al. [11]

An isocratic flow of 0.7 mL/min was used. As mobile phase, ammonia formate (pH 9.5) and, as stationary phase, an XBridge C18 column (5 μm, 4.6 × 150 mm, Waters, Austria) were used. The UV channel was recorded at 195 nm.

### Thin-layer chromatography methods for HEPES quantification

TLC analysis was performed according to the Ph. Eur. for comparison reasons, and the following parameters were evaluated for improving the assay: different spotting methods, stationary phases, mobile phase compositions (Table 2), various forms of iodine staining, and quantification instruments.

### TLC assay according to Ph. Eur. [8]

The Ph. Eur. stipulates for product solutions with a certain maximum volume of administration *V* [mL] a spotting method of “*V*/2000”-times 1-μL droplets of product solution next to a reference solution containing the maximum allowed concentration (see the “Background” section for a more detailed explanation and a model calculation in Fig. 2). The TLC plate was developed in ACN/H_2_O (3/1 *v*/*v*) as mobile phase. After drying, the TLC plate rested in an iodine chamber for 4 min. The two resulting HEPES spots (*R*_*f*_ = 0.3) were compared visually. For release, the product solution spot has to be less or equally intense than the one of the reference solution.

### Schedule for creating a new and improved TLC assay

In order to develop a new TLC assay, the reference solution was prepared in the same composition as the product solution. Afterwards, the spotting volume and spotting method were optimized for a focused HEPES spot that was comparable or even more focused than spotting according to the Ph. Eur. assay. Different mobile phases were tested to obtain an even more focused spot compared to the diffuse spot of the product solution according to the Ph. Eur. All evaluations were performed using a warmed iodine chamber.

### Reference solution

A reference solution was freshly prepared for every product activity concentration containing the maximum allowed HEPES concentration (200 μg/recommended administration volume). The reference solution was further adjusted to the product solution of ^68^Ga-radiopharmaceuticals produced with a Scintomics GRP synthesizer. Therefore, the matrix consisted of PBS/EtOH/H_2_O (16/1/1, *v*/*v*/*v*).

### Sample spotting

For TLC spotting, five different patterns were tested to enhance interpretability of results: 8 μL in 1 μL steps, 8 μL in 2 μL steps, 16 μL in 2 μL steps, 25 μL in 5 μL steps, and 40 μL in 5 μL steps.

### Mobile phase

Ten mobile phases were tested that are listed in Table 1.

**Table 1:** Composition of the tested mobile phases

Mobile phase	Composition (*v*/*v*)
A (Ph. Eur.)	ACN/H_2_O (3/1)
B	ACN/H_2_O/MeOH (15/3/2)
C	ACN/0.25 M NaOAc (3/1)
D	ACN/H_2_O/MeOH (15/5/1)
E	H_2_O/MeOH (1/1)
F	ACN/H_2_O/MeOH (13/3/4)
G	ACN/NaOAc/MeOH (15/3/2)
H	ACN/MeOH (4/1)
I	ACN/H_2_O/MeOH (11/4/3)
J	ACN/MeOH (1/1)

### Optical determination of the HEPES content

Initially, iodine-dyed TLC plates had to be observed visually and the HEPES content was subjectively determined. Judgment whether HEPES was below the allowed limit or not and if the used method was able to give useful information had to be done in a non-standardized manner only based on visual inspection. To improve this procedure, a photo of the TLC plate was analyzed by ImageJ free software to evaluate the contents of HEPES [15, 16]. Therefore, the photo was set to 32 bit. Regions of interest (ROIs) were drawn manually, and the same frame was used across the row. Afterwards, the spots were plotted and the area was measured. Comparison of the ROIs of the HEPES sample and a set of reference solutions enabled standardized relative quantification.

### Evaluation of different methods by determining the HEPES content of a product sample

For the comparison of the tested method, a HEPES-containing product sample synthesized using a Scintomics GRP module was measured using the different methods, i.e., TLC (Ph. Eur.), TLC (improved), and HPLC (Antunes et al.). The TLCs were additionally quantified using ImageJ.

### Evaluation of different TLC methods by using a questionnaire

For evaluation whether the new TLC conditions were more reliable or not, 28 volunteers completed a questionnaire consisting of photographed TLC plates which were performed according to the Ph. Eur. method and the new assay, respectively. Accordingly, a sample with a HEPES concentration unknown to the subjects had to be compared with a spot of a reference solution and determined if the sample contained more or less HEPES than the reference. In this case, a recommended volume of *V* = 8 mL was arbitrarily chosen. Consecutively, the maximum allowed HEPES concentration was calculated as 25 μg/mL.

## Results

In order to implement an HPLC-based quantification method for HEPES in our facility, two literature-known assays were tested for suitability: the methods according to Kvaternik et al. and Antunes et al., respectively [11, 12]. In parallel, the TLC method according to the Ph. Eur. was refined in order to obtain reliable and reproducible results, even when conducted by different operators.

### Comparison of different HPLC assays

**Fig. 3:**
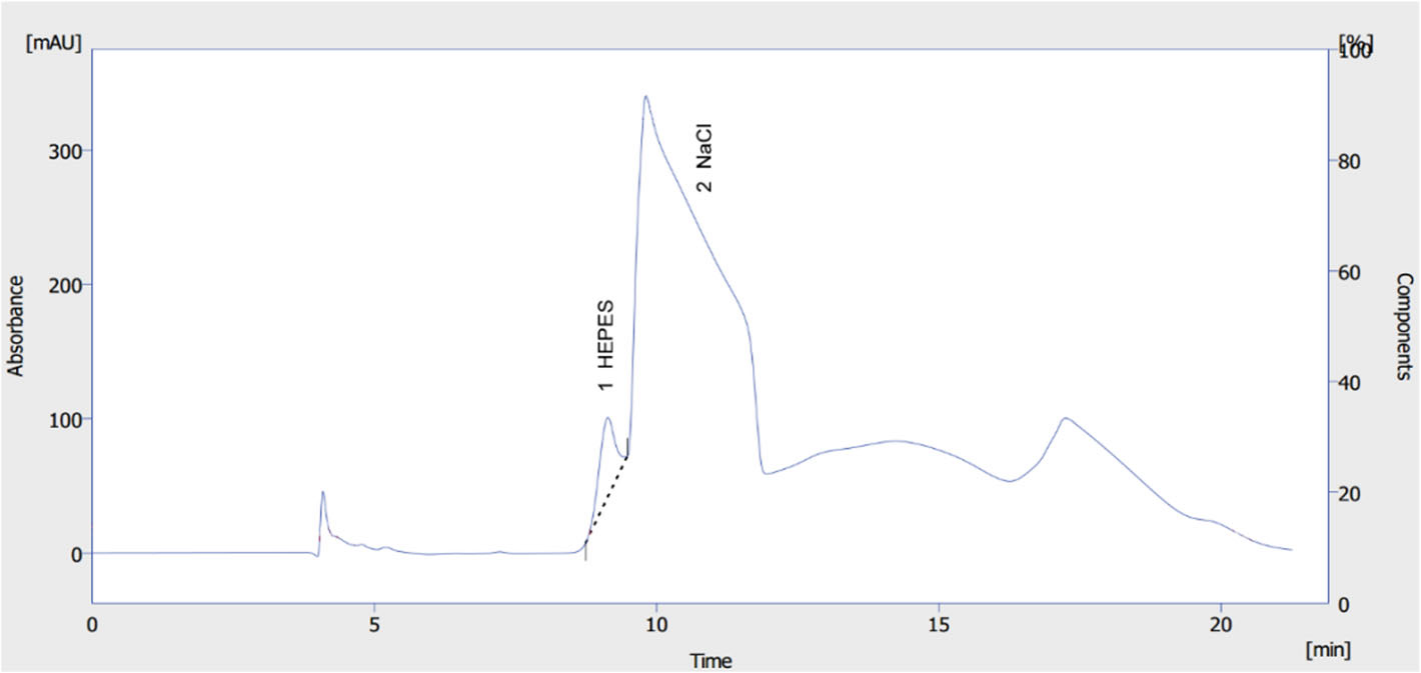
Representative HPLC chromatogram of HEPES (1 mg/mL) obtained by the method according to Kvaternik et al.

The HPLC assay according to Kvaternik et al. [11, 12] was not possible to establish on our VWR Hitachi HPLC system. The VWR Hitachi HPLC device was not capable of separating the HEPES peak from the NaCl peak even if the flow rate was reduced from 0.8 to 0.5 mL/min (Fig. 3).

**Fig. 4:**
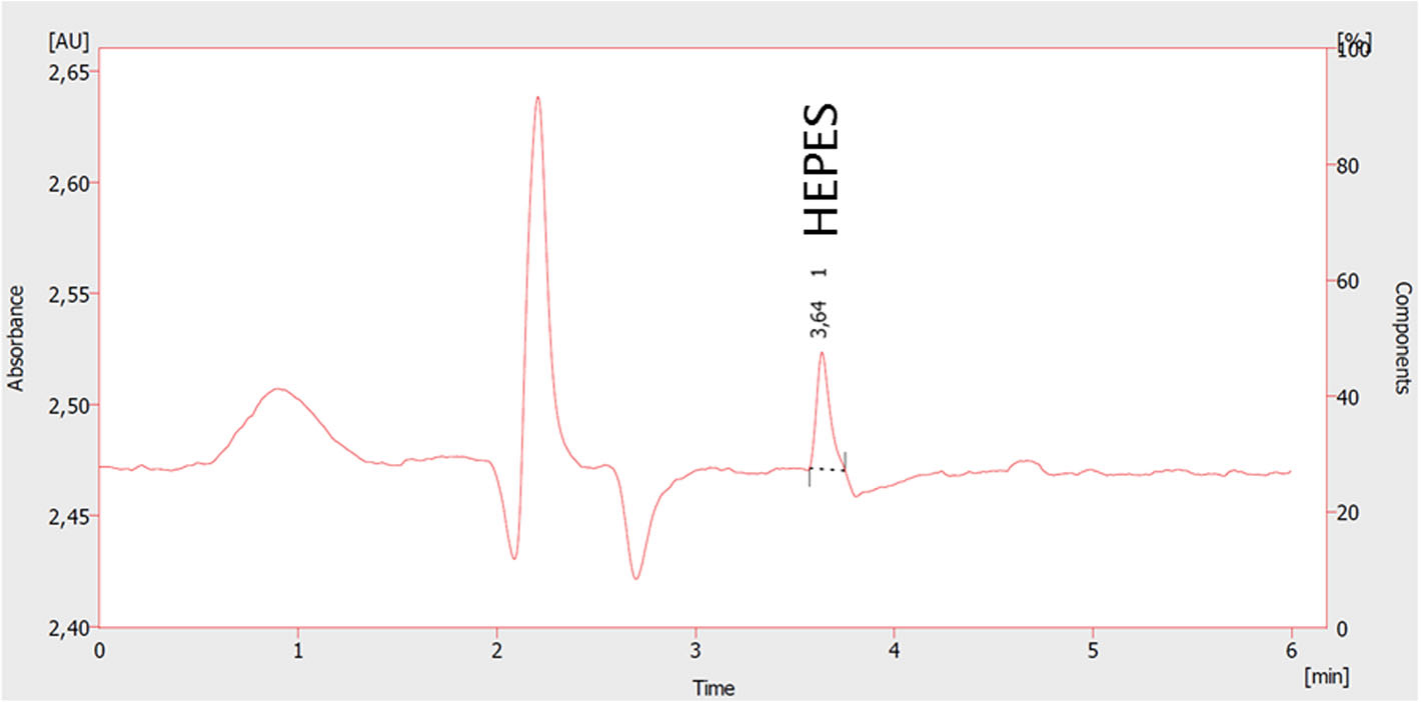
Representative HPLC chromatogram of HEPES (35 μg/mL) obtained by the method according to Antunes et al.

**Fig. 5:**
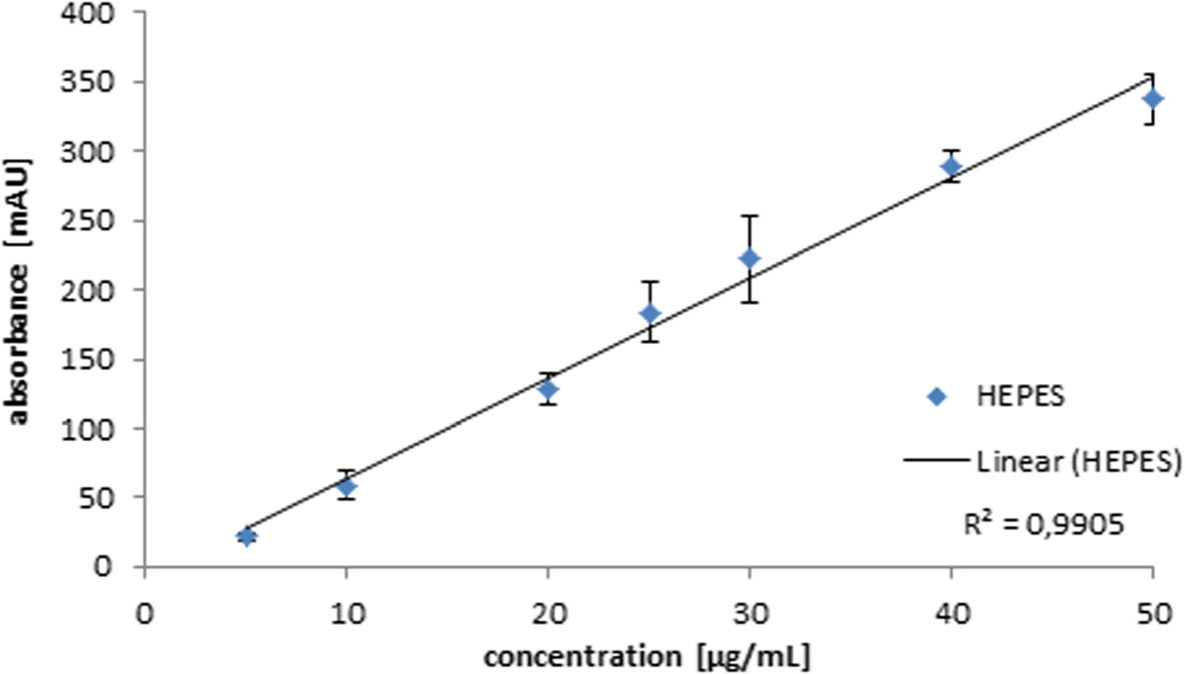
Calibration curve of HEPES applying the HPLC assay according to Antunes et al. (*n* = 3)

The method developed by Antunes et al. could be successfully adapted on the VWR Hitachi HPLC system. A quantifiable HEPES peak was found applying a flow rate of 0.7 mL/min and a mobile phase of 15 mM ammonium formate at pH 9.5. The HEPES peak was baseline separated from all other components of the formulation and quantifiable at a retention time of 3.64 min as depicted in Fig. 4. The respective calibration curve shows a high linearity with an *R*^2^ = 0.9905 (Fig. 5).

### Comparison of different TLC methods

**Fig. 6:**
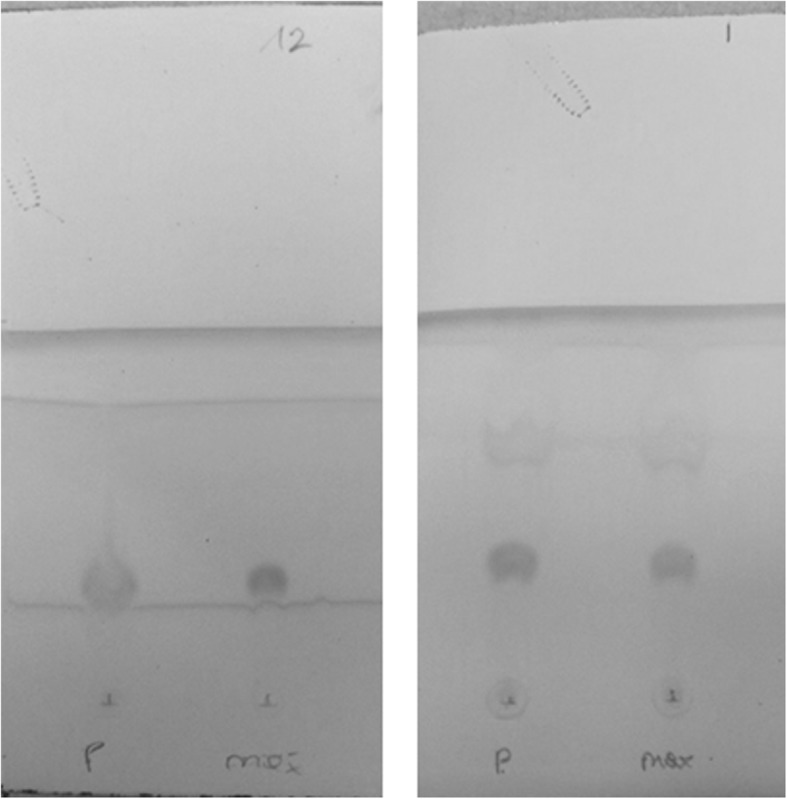
Representative images of iodine-stained TLC plates (35 μg/mL HEPES) with the sample (“P”) spotted in the left column and the maximum allowed concentration (“max,” 25 μg/mL in this experimental setup) in the right column of each plate. The plates were processed according to the Ph. Eur. (left image) and the improved method (right image) and were used for the operator questionnaire

The TLC assay according to the protocol of the Ph. Eur. gave different shapes of iodine spots of product samples (same formulation as the synthesized radiopharmaceutical) and the reference solution (in water). Figure 6 shows that the spot of the reference solution was more focused, whereas the product spot appeared more diffuse. Furthermore, iodine staining of the TLC plate was not reproducible.

Improvement of the TLC method involved the evaluation of different spotting patterns, using the same formulation of the reference standard and sample solution, testing different mobile phases, pre-heating of the iodine chamber, and developing a method as well as image analysis.

Several spotting methods were tested (i.e., 8 × 1 μL, 4 × 2 μL, 8 × 2 μL, 5 × 5 μL, 8 × 5 μL), and results showed that spotting four times 2 μL gave intense spots after staining. For preparation of the reference solution, dissolving HEPES in a solution of the same composition as a clinically used product formulation resulted in a similar shape of the stained spots, but the spot shape was still quite diffuse (see Fig. 6). Accordingly, a new mobile phase consisting of ACN/H_2_O/MeOH (15/3/2) (mobile phase B) was used that resulted in more focused spots and showed a short time of development of less than 10 min. The retention factor (*R*_*f*_) was about 0.35 using this mobile phase. Additionally, warming the iodine chamber to 30–40 °C achieved uniform staining [17].

**Fig. 7:**
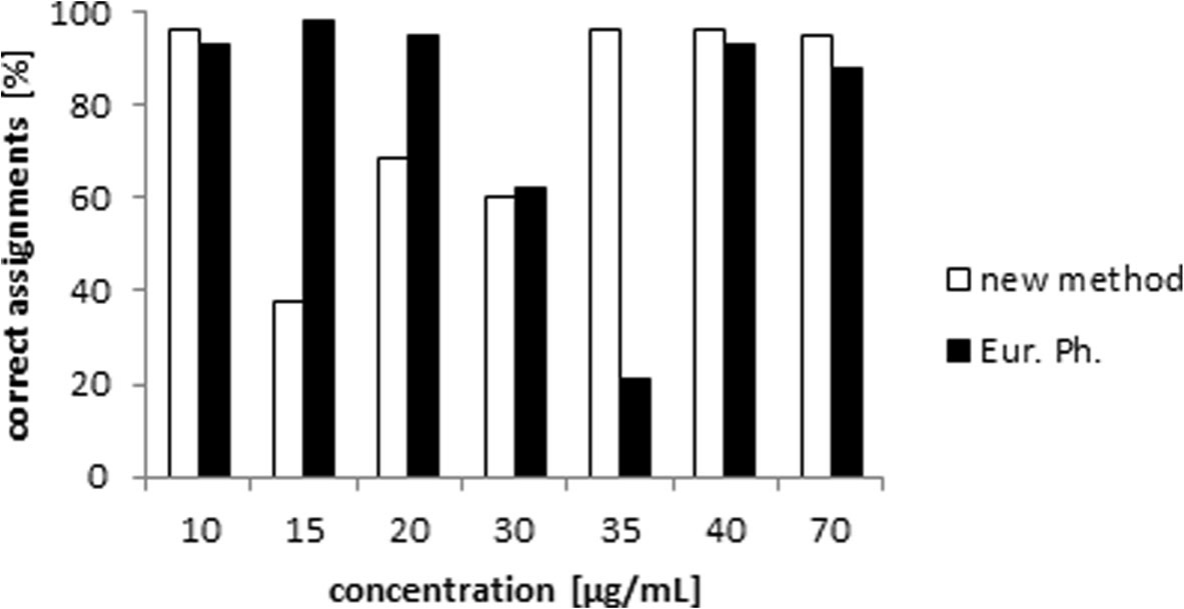
Evaluation of the questionnaires. Correct assignments of HEPES concentration in comparison to the reference solution using visual inspection

The evaluation of the validity of both methods was executed by 28 subjects who filled out a questionnaire that contained photos of a set of TLC strips. These fictive quality controllers had to determine whether or not samples contained more HEPES than a corresponding reference solution. This examination leads to the following results:

- The concentration of HEPES in the solution was underestimated for the method according to Ph. Eur. This resulted in a high rate of false assignments at concentrations of 20 and 30 μg/mL which were above the allowed maximum concentration assumed for a maximum amount of 200 μg in 8 mL (Fig. 7).
- Evaluation of the stained TLC plates was additionally interpreted by means of a pixel-based analysis (using ImageJ software). Applying this quantitative analysis, only at two concentrations (30 and 35 μg/mL, respectively), one false assignment was observed out of two measurements.
- Using the newly established method, the subjects overestimated the HEPES content in the sample at lower concentrations (Fig. 7).
- Pixel-based evaluation of those TLC plates also led to two false results at concentrations 15 and 30 μg/mL for one out of two measurements.

**Fig. 8:**
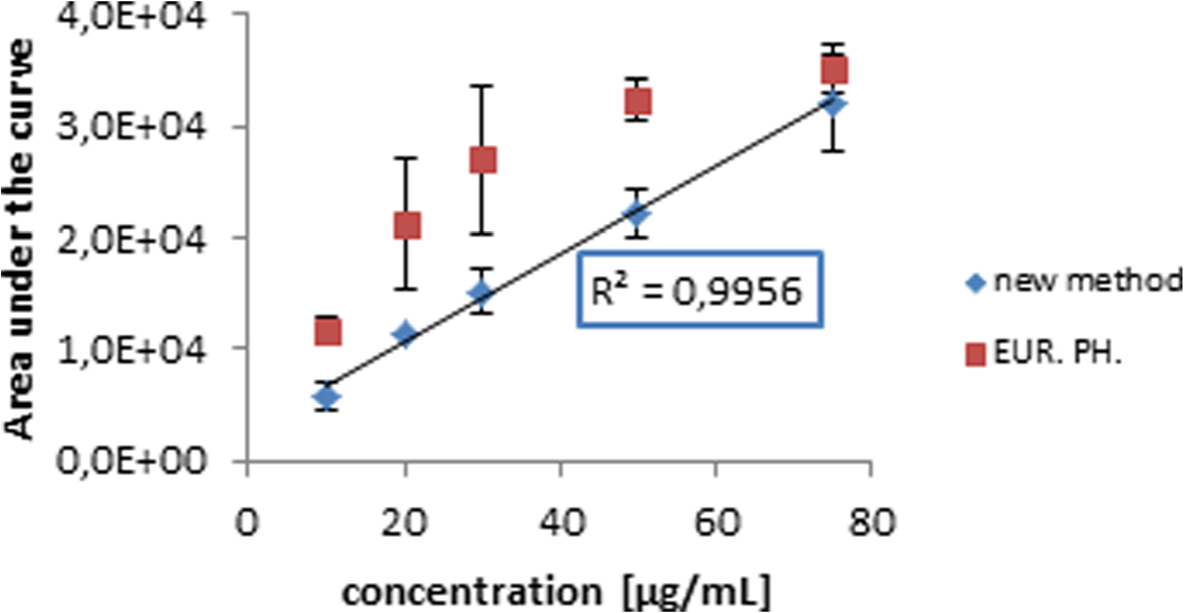
Calibration curves of HEPES concentration obtained from a pixel-based analysis method by means of ImageJ applied to the Ph. Eur. and the improved assay

For both TLC methods, a calibration curve was drawn for the pixel-based analysis. The method for the Ph. Eur. method showed a limit of linearity (LOL) at about 35 μg/mL (Fig. 8). Additionally, values at 20 and 30 μg/mL showed a high standard deviation of 21,210 ± 5875 (28% CoV) and 27,041 ± 6702 (25% CoV) area under the curve, respectively.

TLC analysis using the improved method and ImageJ for evaluation gave a calibration curve with an excellent *R*^2^ = 0.9956 throughout a linear range of 10 to 70 μg.

### Determining the HEPES content of three [^68^Ga]Ga-Pentixafor product samples

The HEPES concentrations determined by HPLC and ImageJ analysis of TLC (improved) gave comparable results for all three product samples (Table 2). In contrast, the ImageJ analysis of TLC (Ph. Eur.) gave only comparable results in accordance with the other methods for product sample 2. The other two measurements using this assay resulted in a concentration deviation of more than 100% without a trend to higher or lower values.

**Table 2:** Model calculation for maximum allowed HEPES concentration and determination of HEPES content in exemplary [^68^Ga]Ga-Pentixafor product samples measured with HPLC and TLC (ImageJ)

Model calculation			
Product activity	Product volume	Activity for administration	Calculated max. HEPES concentration
900 MBq	16 mL	150 MBq (in 2.67 mL)	200 μg/2.67 mL = 75 μg/mL
Determination of HEPES concentration in a radiopharmaceutical			
Method	[^68^Ga]Ga-Pentixafor 1 [μg/mL]	[^68^Ga]Ga-Pentixafor 2 [μg/mL]	[^68^Ga]Ga-Pentixafor 3 [μg/mL]
TLC (**Ph. Eur.**)	**169.4 (no release)**	*23.7*	*2.6*
TLC (improved)	*71.6*	*23.9*	*6.6*
HPLC (Antunes et al.)	*65.97*	*19.17*	*8.83*

For a model calculation of a potential applicable patient dose of 150 MBq and an achieved product activity of 900 MBq in 16 mL, the maximum concentration of HEPES according to Ph. Eur. is 75 μg/mL. Therefore, the standard TLC (Ph. Eur.) resulted in one false negative result, whereas our improved TLC method as well as the HPLC method by Antunes et al. led to release of all three product batches.

## Discussion

### Advantages and limitations of the HPLC method

The HPLC assay according to Kvaternik et al. could not be set up or adapted with a reduced flow of 0.5 mL/min using our HPLC device. Kvaternik et al. described this HPLC assay by applying an Agilent system. In contrast, we used a VWR Hitachi system that was not feasible for a baseline separation of the HEPES peak.

The HPLC method according to Antunes et al. was valid in our laboratory setup and achieved an excellent linearity in a concentration range of 5–50 μg/mL HEPES (Fig. 5) for a reliable HEPES quantification. However, a second HPLC run using the same HPLC system as for routine quality control requires proper pre-conditioning of the column after switching methods. This process including column changing, conditioning, and the HPLC run takes a rather long period of about 30 min that is not feasible for the quality control procedure of short-lived radiopharmaceuticals due to substantial radioactive decay. As an alternative, a second HPLC system for performing two simultaneous runs could be an adequate option. Basically, most radiochemical laboratories are equipped with only one HPLC system for the quality control of radiopharmaceuticals. Therefore, this approach is not implementable in all radiochemical laboratories due to the high effort in equipment. In contrast, every radiochemical laboratory is provided with TLC equipment. Thus, a reliable TLC analysis is definitely preferred to meet the demands of a feasible quality control in terms of a manageable instrumental setup and time efficiency.

### TLC limitations and comparison

The TLC method according to the Ph. Eur. was found to be neither reliable nor fully conclusive. The spots of reference and product solution displayed diverging spot shapes, which made them incomparable. This work showed that this problem originates from different formulations of the HEPES solutions (Fig. 6). The matrix of the reference solution was water while the product solution contained PBS and ethanol. Hence, we refined this method using the same composition of the matrix for both, the reference and the product solution, which resulted in spots with a similar shape. However, the shape was still quite diffuse, which indicated the necessity of a different mobile phase. Among the tested mobile phases (Table 1), the composition of ACN/H_2_O/MeOH (15/3/2) attained highly focused spots after iodine staining (Fig. 6).

Another error source was the iodine chamber that was assumed to contain an inhomogeneous distribution of iodine, which resulted in unreliability and irreproducibility. Thus, the iodine chamber was pre-heated for an even distribution of the gas, which achieved reproducible iodine staining of the HEPES spot. Further changes in the spotting method could facilitate the TLC assay. A spotting method of spotting four times 1 μL gave a relatively weak staining according to the low amount of HEPES in 4 μL and was prone to pipetting errors. If the recommended application volume is more than 8 mL with regard to the activity of the generator and, accordingly, the product, the spotting (V/2000 mL in 1 μL steps, see Fig. 2) is time consuming, especially considering that a maximally focused spot had to be achieved by letting the spotted microliters dry before the next one was added. Therefore, using this method for the radioactive probe is not ideal in terms of radiation protection and decay of the product.

On the basis of the high number of error sources, a low validity and reproducibility of the assay according to Ph. Eur. was not surprising. However, this TLC method and subsequent evaluation by comparative, visual quantification is still a semi-quantitative method, which is worthwhile to be substituted by quantitative analysis. Eventually, this could be managed by application of a pixel-based analysis by means of ImageJ. As a result, errors caused by subjective evaluation and inter-observer variability are potentially erased. For evaluations of the TLC methods using ImageJ analysis, a calibration curve with an impressive *R*^2^ of 0.9956 was created for the newly developed TLC assay, in contrast to the calibration curve of the TLC method according to Ph. Eur. in which it was not possible to assign a reasonable linearity. Those results confirm that a refinement of the TLC assay according to Ph. Eur. is of imperative urgency.

For evaluation, all three methods were compared in a realistic setting: Three samples of [^68^Ga]Ga-Pentixafor obtained from routine production were analyzed by HPLC and both TLC methods. The HPLC assay by Antunes et al. and the improved TLC method resulted in comparable HEPES concentrations, whereas the TLC method according to Ph. Eur. was only for one sample in a comparable range with the other methods.

A correct assignment of the HEPES content determined by HPLC and the new TLC method seems more reliable and reproducible than the method proposed in Ph. Eur. However, in clinical routine, it is not necessary to determine the exact HEPES content; a limit test to determine whether or not the HEPES amount in the sample is below the maximum limit is sufficient. Therefore, a direct comparison with the maximum concentration provides sufficient information. For evaluation, a questionnaire involving 28 subjects was set up providing information about the feasibility of the assay according to Ph. Eur. and the improved method when the sample solution is spotted next to the reference solution with the maximum concentration. The HEPES content according to the Ph. Eur. method was basically underestimated, which facilitated the release of radiopharmaceuticals that might fail the required quality criteria. In contrast, many subjects overestimated the HEPES concentration using the new TLC method, which provided an improvement in terms of inherent safety. On the other hand, a radiotracer that is in accordance with the guidelines may not be released in this case. To overcome this inter-observer variability, a pixel-based evaluation of the TLC plates using ImageJ software was introduced and proved to be reliable and time effective.

Therefore, the optimum setup for quick results consists of a TLC analysis applying the improved method and performance of a visual evaluation. The product sample can directly be spotted on a TLC strip next to the reference solution. If the HEPES spot is not clearly distinguishable from the reference spot of 200 μg/*V*, a subsequent pixel-based analysis is recommended, which takes about 3 min. Overall, the novel procedure offers a significant improvement to the available methods for HEPES testing.

## Conclusion

In the present work, different available methods for the determination of HEPES content in radiopharmaceutical formulations were evaluated. It was shown that the TLC assay according to Ph. Eur. was neither reliable nor reproducible to determine the HEPES content in radiopharmaceutical product formulations in our laboratory setting. Since a previously presented HPLC method gave satisfactory results, but required a high additional technical effort, we developed an improved, simple, and valid TLC assay that proved its feasibility for visual evaluation and additional software-based, quantitative analysis. In summary, the newly developed TLC method facilitates HEPES determination substantially and is worth to replace the TLC assay proposed in Ph. Eur. for a straightforward evaluation of the HEPES content.

## Abbreviations

ACN: Acetonitrile
CoV: Coefficient of variance
HEPES: 4-(2-Hydroxyethyl)-1-piperazineethanesulfonic acid
HPLC: High-performance liquid chromatography
NaOAc: Sodium acetate
PET: Positron emission tomography
Ph. Eur.: European Pharmacopoeia
TLC: Thin-layer chromatography
